# FROST: Fallback Voice Apps Recommendation for Unhandled Voice Commands in Intelligent Personal Assistants

**DOI:** 10.3389/fdata.2022.867251

**Published:** 2022-04-25

**Authors:** Qian Hu, Thahir Mohamed, Wei Xiao, Xiyao Ma, Xibin Gao, Zheng Gao, Radhika Arava, Mohamed AbdelHady

**Affiliations:** Amazon Alexa AI, Seattle, WA, United States

**Keywords:** intelligent personal assistants, recommender system, deep learning, paraphrase detection, data relabeling

## Abstract

Intelligent personal assistants (IPAs) such as Amazon Alexa, Google Assistant and Apple Siri extend their built-in capabilities by supporting voice apps developed by third-party developers. Sometimes the smart assistant is not able to successfully respond to user voice commands (aka utterances). There are many reasons including automatic speech recognition (ASR) error, natural language understanding (NLU) error, routing utterances to an irrelevant voice app, or simply that the user is asking for a capability that is not supported yet. The failure to handle a voice command leads to customer frustration. In this article, we introduce a fallback skill recommendation system (FROST) to suggest a voice app to a customer for an unhandled voice command. There are several practical issues when developing a skill recommender system for IPAs, i.e., partial observation, hard and noisy utterances. To solve the partial observation problem, we propose collaborative data relabeling (CDR) method. To mitigate hard and noisy utterance issues, we propose a rephrase-based relabeling technique. We evaluate the proposed system in both offline and online settings. The offline evaluation results show that the FROST system outperforms the baseline rule-based system. The online A/B testing results show a significant gain of customer experience metrics.

## 1. Introduction

Intelligent personal assistants (IPAs) such as Alexa, Siri, and Google Assistant have been becoming more and more popular and making people's daily lives convenient. IPAs can fulfill users' request by answering questions ranging from weather to stock price. To enrich the user experience, a large amount of third-party (3P) voice apps (aka skills) have been developed. These voice apps extend IPAs built-in capabilities to better serve customers. They can perform operations such as ordering food, playing a game, or helping a user sleep by playing soothing sounds. The supported 3P skills can number up to hundreds of thousands.

**Figure 1:**
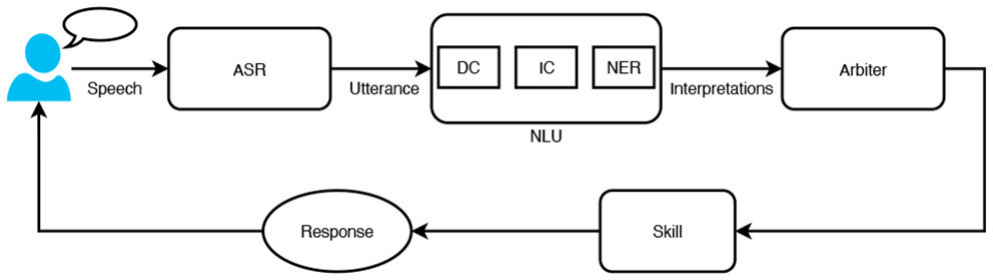
A high-level overview of an intelligent personal assistant (IPA).

Intelligent personal assistants understand user's request using spoken language understanding (SLU) system. The request goes through a series of components to get a response, as illustrated in Figure 1. The first component is automatic speech recognition (ASR), which converts speech to its transcription also called utterance. At the second stage, the utterance is interpreted by the natural language understanding (NLU) system. NLU as the critical component of SLU interprets the meaning of an utterance by using several natural language processing (NLP) technologies including domain classifier (DC), intent classifier (IC), and named entity recognition (NER). The DC determines which domain should process the request. The IC predicts what the user wants to do from the list of intent types of the identified domain. NER, or slot tagging, finds the entity (i.e., person, place, or thing) in the utterance and tags it as a particular entity type (i.e., city, song). For example, given an utterance “play Million Reasons by Lady Gaga.,” the DC predicts Music as the domain. The IC predicts the intent as PlayMusic. Finally, NER identifies the slot-value pairs as SongName:Million Reasons and Artist:Lady Gaga. After NLU, the arbiter is responsible to select the most relevant voice app (skill) for a given NLU interpretation { Music, PlayMusic, SongName:Million Reasons, Artist:Lady Gaga}. Sometimes the arbiter may fail to find a relevant skill that can handle the user request. It could be a system error such as ASR error, NLU error. Another reason could be that the feature requested by the user is not supported yet by the dialog system or the requested content is not found such as music, video, book, and recipe. To reduce customer friction and recover the conversation, we propose a skill recommender system that proactively suggests 3P skills to users for unhandled requests, even if the users are not aware of the skills.

**Figure 2:**
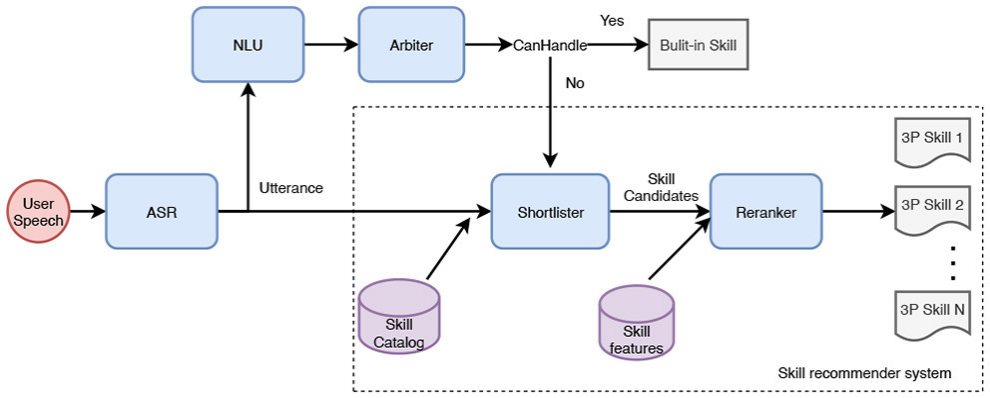
A overview of skill recommender system.

The proposed skill recommender system is composed of two components: a shortlister, and a reranker. Figure 2 shows the system architecture. Given an utterance, shortlister, also known as the candidate generator retrieves *k* most relevant skills out of the skill catalog. The skill catalog for a certain locale has tens of thousands of voice apps and is continuously changing as skills are added, deleted, or updated. This stage is optimized to have a high recall. The retrieved skills are passed to the reranker that ranks the skill candidates by using skill specific information and utterance. Finally, the top-1 skill is presented to users. The advantage of adopting multiple-stage architecture is to improve the recommendation inference time. The first stage depends on simple input to quickly reduce the number of candidates from tens of thousands to *k*. The second stage uses more complicated model architecture and more skill-related features without suffering from complexity issues as it works on a very small number of candidates. This system is not meant to replace the original NLU or arbiter components. It is specifically designed to serve as a fallback for utterances that are not handled by the existing system (i.e., unclaimed utterances) using the increasing catalog of 3P skills.

Traditional recommender systems such as video recommendation recommend a ranked list of items to a user. The user scans the list and selects the one they like the most (Covington et al., 2016). The feedback from the user is treated as the label (accept/reject) for learning a model. However, due to the limitation of the voice user interface (VUI), we can only present the top-1 skill to users, as listening to the playback of a long list is tedious and can significantly degrade user experience (Cohen et al., 2004). This limitation results in a partial observation problem. Namely, users cannot observe the full recommendation list and make a decision, which imposes difficulties in learning a ranking model. To solve the partial observation problem, we propose a novel method called collaborative data relabeling (CDR). CDR mitigates the partial observation problem by trying to answer a counterfactual question, "what if we present another skill to the user?". CDR answers this question by matching a similar request and using feedback from that request to relabel the original ranked list. User feedback is noisy, which results in noisy labels that can hurt the performance of machine learning models. CDR also allows us to mitigate these noisy labels by using multiple similar requests to determine the label. Recommender systems usually focus on optimizing the accuracy of predictions while ignoring the diversity of recommended items, which can degrade user experience if similar items get recommended over and over again (Ziegler et al., 2005; Knijnenburg et al., 2012; Castagnos et al., 2013; Ekstrand et al., 2014; Willemsen et al., 2016). CDR improves the diversity of recommended skills by relabeling different skill candidates that serve the same intent. The relabeled skills force the model to learn to diversify its prediction distribution among multiple skill candidates.

Another challenge of building a skill recommender system is noisy and hard utterances that cannot be handled by NLU. Customers express their intents in many different ways with a long tail of rare utterances (Falke et al., 2020) which are hard for the voice assistant to interpret. Although these utterances are rare, in aggregate their volume is huge. Besides hard utterances, the input utterances can be noisy and erroneous due to background noise and ASR errors. For noisy and hard utterances, it is hard for the voice assistant to interpret them. However, we found that customers often try to help voice assistant by rephrasing their utterances until it understands them. To make the model learn to handle hard and noisy utterances, we propose rephrase-based relabeling. This method identifies the rephrased utterances of the unclaimed utterances within the same session that are handled successfully by the voice assistant. Then it uses the invoked skill of the claimed rephrase utterance as a ground truth label of the unclaimed utterance.

In the beginning, we do not have data for training the model. To collect training data, we build a rule-based system. Similar to the proposed system, the rule-based system also has a two-stage architecture. We use the data collected from this system to train and evaluate our proposed model offline. The proposed model is put into production for online A/B testing after it has achieved satisfying offline results. Online experimental results show significant gains of user experience metrics such as higher volume of acceptances and lower friction rates.

Overall, the contributions of this study are summarized as follows:

- We propose a skill recommender system for IPAs to handle unclaimed utterances by exploiting the ever-increasing 3P voice apps.
- To mitigate the partial observation issue, we propose CDR inspired by causal inference. CDR has the additional advantage of solving noisy label issues.
- Collaborative data relabeling also has the advantage of improving recommendation diversity and, thus, improving user satisfaction. Suggesting diverse skills to users can help them explore and discover more skills, which is also beneficial to third-party skill developers.
- To make the model robust to hard and noisy utterances, we propose rephrase-based relabeling. This method utilizes customer paraphrasing behavior to help the model learn to handle noisy and hare utterances.
- We conduct offline and online experiments. Online experimental results show significant gains in user experience metrics.

## 2. Skill Recommender System

Our skill recommender system consists of two components, shortlister and reranker, as shown in Figure 2.

### 2.1. Shortlister

Given the input utterance text, the shortlister selects top-*k* relevant skills from the skill catalog. We implement shortlister as a keyword-based search engine. To build the skill search engine, we index skill metadata including skill name, skill descriptions, example phrases, and invocation phrases. At retrieval time, the relevancy score between an utterance and a skill is computed as the sum of TF-IDF score (Rajaraman and Ullman, 2011) of every word in the utterance. The skills with top *k* relevancy scores are returned. Shortlister plays an important role as it decides the performance upper bound of the whole system. One of the benefits of using search-based skill retrieval is that we do not need to use customer feedback to train the shortlister component, which is especially helpful at the beginning of the development of the system as we do not have any customer feedback data. However, since it uses a keyword-based matching technique, the semantic meaning of the utterance is ignored. We use the reranker to rank the skill candidates generated by shortlister to make sure the most relevant skills are ranked at the top.

### 2.2. Reranker

**Figure 3:**
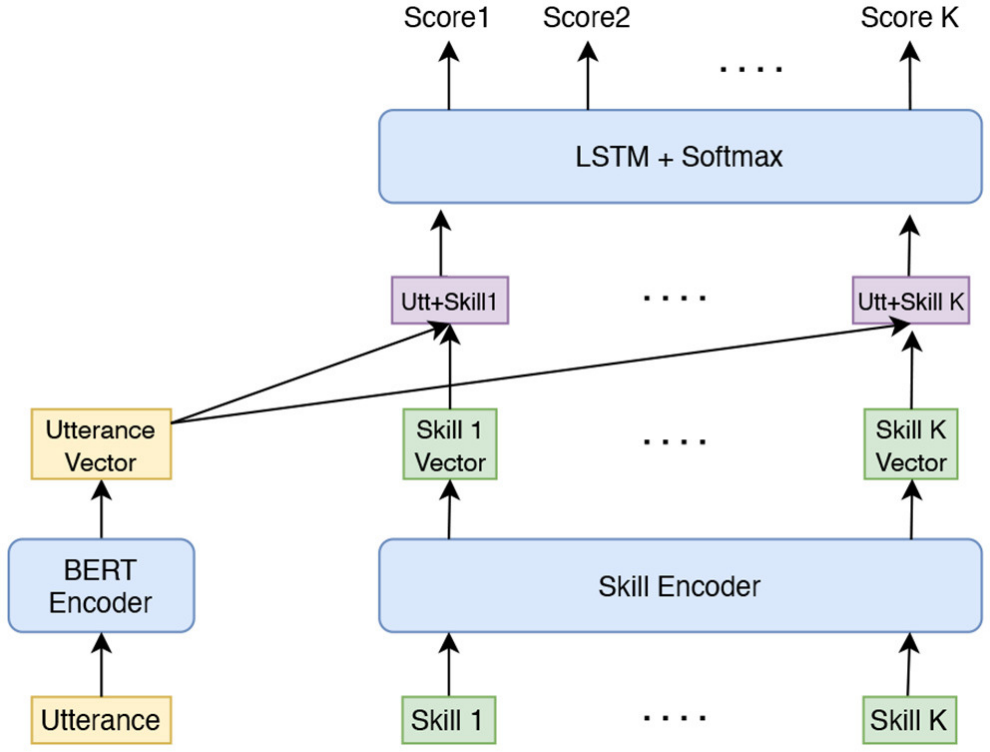
The model architecture of reranker.

The reranker model takes in the skill candidates generated by shortlister and returns a ranked list of skills based on utterance and skill specific information. The reranker is a deep learning model with a listwise ranking loss function. Figure 3 shows the reranker model architecture. The utterance is encoded by a Bidirectional Encoder Representations from Transformers (BERT) encoder (Devlin et al., 2018). The features of skills include skill id, skill name, and skill score returned by shortlister. Skill id is represented using an embedding vector; skill name is encoded into an embedding vector by BERT. The skill score feature is converted into a bin and encoded as an embedding vector. The skill feature embedding vectors are concatenated to form a single embedding vector. The utterance embedding vector is concatenated with every skill embedding vector to form a sequence of utterance-skill embedding vectors. As the skill candidates returned by shortlister is ordered by relevance score, to capture such sequential information, these sequence of embedding vectors are put into a Bi-LSTM layer (Hochreiter and Schmidhuber, 1997). The outputs from the Bi-LSTM layer are converted to probability scores by using the softmax function. Each skill has a corresponding probability score. The skills are reranked according to the predicted probability scores.

When suggesting a skill to a user, the voice assistant asks the user if they want to accept it. A skill is launched if the user agrees to accept it, which is regarded as a positive label; otherwise, the label is negative. For a list of skill candidates, the user feedback for the skill candidates is **y** = {*y*_1_, …, *y*_*k*_}, *y*_*i*_∈{0, 1} and the predicted probabilities by the reranker model is **s** = {*s*_1_, …, *s*_*k*_}. We use the listwise ranking objective function (Cao et al., 2007). The objective function is formulated as


(1) $$L\left(y,s\right)=\frac{1}{k}\sum_{i=1}^{k}\left[-y_{i}\log\left(s_{i}\right)-\left(1-y_{i}\right)\log\left(1-s_{i}\right)\right].$$

Compared to pointwise (Shashua and Levin, 2003) and pairwise (Burges, 2010) rerankers which treat each skill independently, listwise reranker models the whole list so that it can capture the correlation between them. Due to the partial observation issue, the labels of the unobserved skills are treated as negative. However, this assumption is not realistic and can bias the model, because the missing values are not necessarily negative. In the next section, we introduce CDR to mitigate this issue. Another advantage of using the listwise model is efficiency. The pointwise model needs to encode the utterance *k* times, while the listwise model only needs to encode the utterance once. Online experiments also show that the listwise model reduces latency significantly.

## 3. Collaborative Data Relabeling

Compared to traditional recommender systems such as video recommendation where users view the full recommended list and select the best one they like, the skill recommender system has its unique challenge. Limited by VUI, we can only present the top-1 ranked skill to the user, which results in a partial observation problem. With partial observation, users have no chance to view and compare other skills in the list. We do not know if the user would like the other skills more than the top-1. Without comparing the top-1 skill with the other skills, it is hard to learn a ranking model, as ranking, in essence, is about comparing. To solve the partial observation problem, we propose CDR approach.

**Figure 4:**
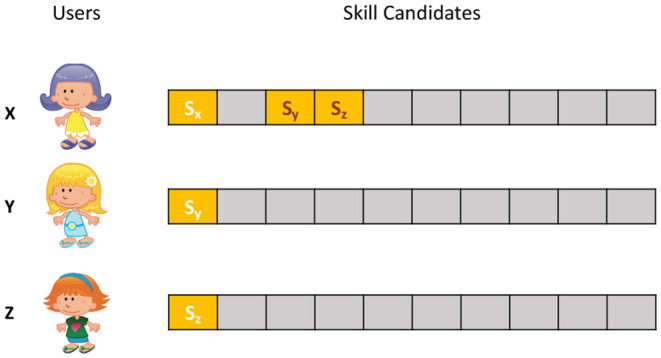
Illustration of collaborative data relabeling (CDR). In skill recommendation, only the top-1 skill is presented to users. Given a user x who invoked voice assistant with some utterance, to know her responses to skills s_y_ and s_z_ that were not presented to her, we found two users y and z who spoke similar utterances and were suggested with skills s_y_ and s_z_, respectively, and use their responses to relabel user x's feedback to skills s_y_ and s_z_.

The intuition of CDR is to answer a counterfactual question, namely, “what if we had presented another skill to the user?”. To answer this question, we find *k* nearest neighbors of a user request (utterance) and use their feedback to relabel the original ranked list of skill candidates, which is inspired by matching method (Stuart, 2010) in causal inference. In causal inference, matching is an approach to estimate the treatment effect of a treatment by comparing the treated units to non-treated units with similar characteristics. CDR has a similar working mechanism. Given a user utterance, to know the user's response to an unpresented skill, we find a similar utterance whose invoker has interacted with that skill and used their response to relabel it as either positive or negative, as illustrated in Figure 4. Usually, there are more than one neighbors that have interacted with the skill, in which case we use majority vote to decide the final label.

### 3.1. Handling Noisy Labels

Customer feedback is noisy, which results in noisy labels. As the suggested skill is presented to the user by reading its name, in some cases, the skill name might mislead the user to accept it but cannot fulfill the user request, which results in a false positive label. Noisy labels can severely hurt the model's generalization performance. Another source of noisy labels is from the neighbors at the relabeling stage. Some of the neighbors might not be similar to the source utterance, which introduces noisy labels when relabeling the source skill candidates. Another advantage of CDR is its ability to mitigate the impact of noisy labels. The intuition is that instead of using one user's response to a skill as the label, we choose several similar users and aggregate their responses to a skill by majority vote. As the final decision is made by several users, the label is more reliable than that of just one user.

### 3.2. Recommendation Diversity

Recommender systems are confronted with an over-fitting problem that only a small portion of items are recommended to users (Kunaver and Požrl, 2017), which can hurt user satisfaction as they can quickly get bored by always being suggested with similar types of items. This problem is especially relevant for skills that serve the same intent with different content. For example, when users ask to play a soothing sound to help them sleep, always suggesting the same sleep sound can get users bored, while there exist many types of sleep sounds in the skill store such as frog, ocean, rain, and waterfall sleeping sound. Suggesting diverse skills can improve user satisfaction (Castagnos et al., 2013). The proposed CDR method improves diversity by relabeling different skill candidates as positive, which forces the model's predictive distribution to be dispersed among more skills. Diversified suggestions can lead to a drop in accuracy (Ziegler et al., 2005; McNee et al., 2006), which imposes difficulties in faithfully evaluating the real user satisfaction metrics. To evaluate how diversity can influence user satisfaction, we use manual annotation. The detail of the manual annotation schema will be explained in Section 6.1.2.

### 3.3. Interpretability

Collaborative data relabeling relabels a skill candidate by using the response of a neighboring utterance. The proposed CDR is interpretable in the sense that when relabeling a skill candidate, we know why it gets relabeled by inspecting the neighboring utterance. The benefit of interpretability is that if the neighboring utterance does not make sense, we can discard the corresponding relabeled label.

### 3.4. Similarity Metrics

The core part of CDR is similarity measurements between two utterances. We investigate several approaches for measuring similarities.

#### 3.4.1. Jaccard Similarity

Given an utterance, the output from the shortlister is *k* skill candidates. If two utterances are similar, their corresponding skill candidates should also be similar. Therefore, we can measure the similarity between utterances by comparing their skill candidates. For utterances *i* and *j*, their skill candidates are sc_*i*_ and sc_*j*_. The similarity score between utterances *i* and *j* is Jaccard similarity between sc_*i*_ and sc_*j*_


(2) $$\begin{array}{r} \text{sim}\left(i,j\right)=\frac{{\text{sc}}_{i}\cap {\text{sc}}_{j}}{{\text{sc}}_{i}\cup {\text{sc}}_{j}}. \end{array}$$

#### 3.4.2. Term Frequency and Inverse Document Frequency (TF-IDF)

We treat each utterance as a document and compute the term frequency and inverse document frequency of each word in an utterance. Each utterance is represented as a vector. An entry in the vector represents the TF-IDF (Rajaraman and Ullman, 2011) value of the corresponding word. The similarity score of two utterances is computed as the cosine similarity of their vectors.

#### 3.4.3. Semantic Similarity

To capture the semantic meaning of utterances for similarity measurements, we use fine-tuned BERT encoder to encode utterances into embedding vectors. The BERT model is fine-tuned using data with a multi-task objective function, specifically, intent classification, and NER. We also experiment with a pre-trained BERT encoder and find that it does not work well for capturing the semantics of an utterance, which has also been discovered by several works such as Reimers and Gurevych (2019) and Li et al. (2020). We use the average pooling of the contextual embedding vectors in the last layer as the utterance embedding vector. We measure the similarity score between two utterances using cosine similarity between their embedding vectors.

## 4. Rephrase Based Relabeling

Noisy and hard utterances can negatively impact the performance of SLU. This is especially problematic for the fallback skill recommender system (FROST) as utterances that cannot be handled by the voice assistant come to the fallback component. To build a successful skill recommender system, it needs to handle hard and noisy utterances. We found that when the voice assistant does not understand them, users tend to rephrase what they said to help it. The rephrases are usually simpler and the voice assistant is able to provide correct answers. To build a skill recommender system that is robust to hard and noisy utterances, we propose rephrase-based relabeling, which utilizes labels from simpler utterances to label hard and noisy utterances.

The critical part of rephrase-based relabeling is rephrase detection. Given the original utterance with friction, we want to identify the utterance within the same session that is the rephrasing of the original utterance and successfully served by the voice assistant.

### 4.1. Rephrase Detection

Given an unclaimed utterance, the task of rephrase detection is to find an utterance after *u*_*t*_ in the conversation session that is semantically similar to the unclaimed utterance *u*_*t*_. The assumption is that the users tend to rephrase their requests in the conversation with the voice assistant until they get the expected response. A session is a sequence of utterances denoted as {*u*_1_, …, *u*_*t*_, .., *u*_*N*_}. Assume *u*_*t*_ is the original utterance, we want to find rephrase in {*u*_*t*+1_, …, *u*_*N*_}. An utterance is considered as a rephrasing of the original utterance if they are semantically similar. To measure the similarity of two utterances, we encode the utterances into embedding vectors using a pre-trained BERT encoder as in Section 3.4.3 and the similarity score is computed as the cosine similarity between the two embedding vectors. A future utterance is regarded as the rephrase of the original utterance if its similarity score is above a certain threshold. We decide the optimal similarity threshold by manually checking the original and rephrase pairs.

### 4.2. Relabeling

Given an unclaimed utterance *u*_*t*_, its conversation session {*u*_1_, …, *u*_*t*_, .., *u*_*N*_} and skills that served the corresponding utterances {*l*_1_, …, *l*_*t*_, …*l*_*N*_}, the rephrase-based relabeling works as shown in Algorithm 1.

**Algorithm 1:**
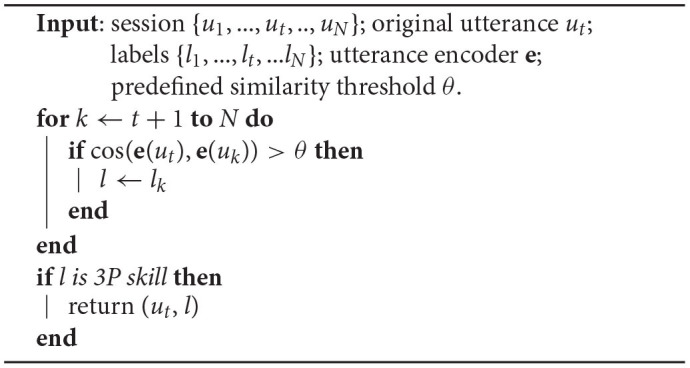
Rephrase-based relabeling.

Note that we treat the last similar utterance within the session as the rephrase and we only relabel the original utterance if the rephrase is served by a 3P skill.

## 5. Experiments

### 5.1. Data Collection

In the beginning, we do not have data to train and evaluate our system. To collect data, we build a rule-based system that has similar architecture as our proposed one. The rule-based system uses the same shortlister but a different reranker. The difference is the reranker. The input into the rule-based reranker is the skill candidates from shortlister. The rule-based reranker ranks the skill candidates by using their historical acceptance rates. The skill with the highest acceptance rate is selected. To ensure high quality of recommendation, we only suggest the top skill to the customer if its acceptance rate is higher than 0.5. We collect 2-month data from a commercial voice assistant traffic for model training and evaluation. The data of the last week is used for testing. The data of the second last week is used for validation. The remaining is used for training. The proportions of training, validation, and testing data are around 80, 10, and 10%, respectively. Each data sample is composed of an utterance *u*_*t*_, forty skill candidates *s*_*t*, 1_, …, *s*_*t*, 40_ generated by shortlister, and ground truth label *y*_*t*_, denoted as (*u*_*t*_, (*s*_*t*, 1_, …, *s*_*t*, 40_), *y*_*t*_), where *y*_*t*_∈{*s*_*t*, 1_, …, *s*_*t*, 40_}∪{*N*}, *N* is null which means all the skill candidates are rejected by the user. The features of a skill *s*_*t*, 1_ include skill id, skill name, and skill score from shortlister; skill id is represented as one-hot vector and skill score is quantized into one of three levels (high, medium, and low) and represented as one-hot vector as well. Note that for the sake of customer privacy, the data is de-identified and we are not able to know the identity of the user from the data.

### 5.2. Collaborative Data Relabeling

For CDR, the *k*-nearest neighbors of an utterance are found from the training data. Additionally, only the training data is relabeled. We keep the labels of the validation and testing data as it is. When relabeling the skill candidates of an utterance, we select up to 200 neighbors and keep those whose similarity score is above a certain threshold *s*. To avoid bringing noisy labels from neighbors, a skill candidate is relabeled if the number of its supportive neighbors is higher than *n*. The supportive neighbors of skill are the neighboring utterances that relabel it as positive. The intuition is that if there are multiple neighbors confirming a skill candidate, the relabeled skill is reliable. We treat *s* and *n* as hyperparameters and choose the best value by using the validation dataset.

**Figure 5:**
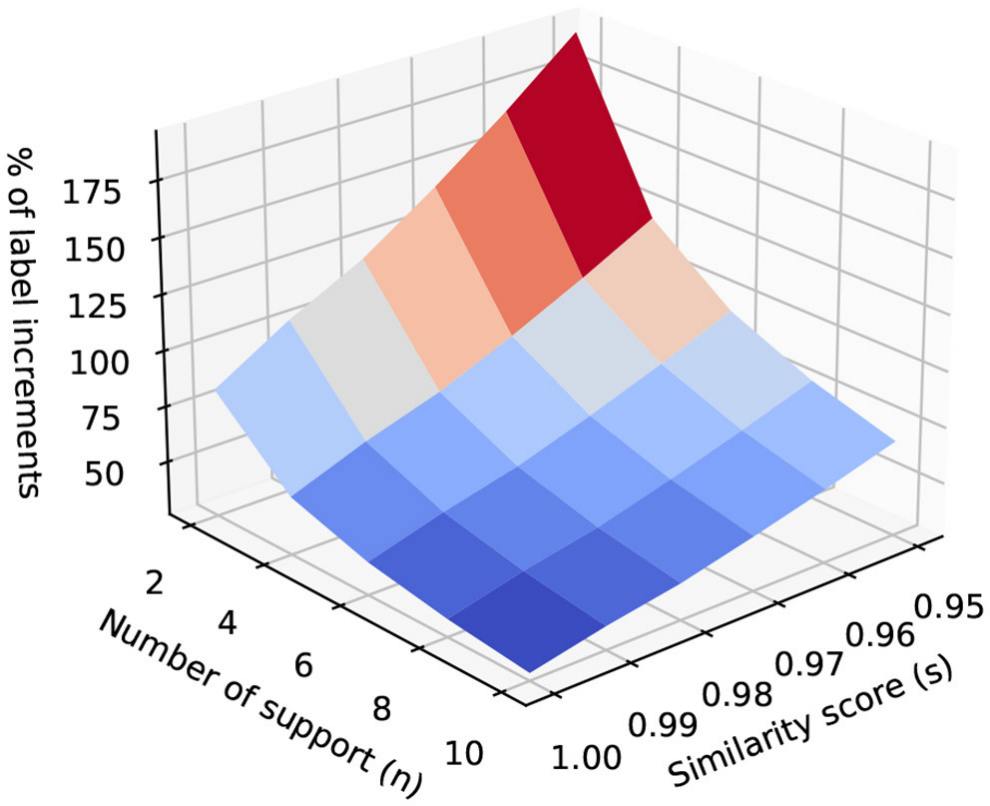
The percentage of label increments after applying CDR.

Figure 5 shows the percentage of label increments after applying CDR. In this figure, we use cosine similarity between utterance embedding vectors based on fine-tuned BERT model, refer to Section 3.4.3. It shows that with the decreasing of the similarity score threshold, the number of relabeled labels increases. When the number of support increases, the number of relabeled labels decreases as it requires more neighbors to support a skill to be relabeled.

### 5.3. Evaluation Metrics

Given a list of skill candidates, the reranker ranks them and returns a ranked list of skill candidates. The top-1 skill with a predicted probability higher than 0.5 is presented to a user. If the predicted probability of the top-1 skill is lower than 0.5, no skill is suggested. To simulate this scenario, we evaluate the model by selecting the top-1 skill with a predicted probability higher than 0.5 and comparing it with the ground truth label. The evaluation metrics we use include precision, recall, and F1 score. Given the ground truth labels {*s*_1_, …*s*_*i*_, …, *s*_*N*_} and predicted labels {ŝ_1_, …, ŝ_*i*_, …, ŝ_*N*_}, *s*_*i*_, ŝ_*i*_∈*S*∪{*N*}, where *S* denotes all the skills, the evaluation metrics are defined in Table 1. Precision_1_ calculates the number of correct predictions over all the predictions, while Precision_2_ means the number of correct predictions over all the utterances that have non-empty ground truth labels. In this article, we report relative performance.

**Table 1:** Evaluation metrics.

**Metrics**	**Equation**
Precision_1_	$\frac{\underset{i}{\sum }1\left(s_{i}={ŝ}_{i}\right)\cdot 1\left(s_{i}\neq N\right)\cdot 1\left({ŝ}_{i}\neq N\right)}{\underset{i}{\sum }1\left({ŝ}_{i}\neq N\right)}$
Precision_2_	$\frac{\underset{i}{\sum }1\left(s_{i}={ŝ}_{i}\right)\cdot 1\left(s_{i}\neq N\right)\cdot 1\left({ŝ}_{i}\neq N\right)}{\underset{i}{\sum }1\left(s_{i}\neq N\right)\cdot 1\left({ŝ}_{i}\neq N\right)}$
Recall	$\frac{\underset{i}{\sum }1\left(s_{i}={ŝ}_{i}\right)\cdot 1\left(s_{i}\neq N\right)\cdot 1\left({ŝ}_{i}\neq N\right)}{\underset{i}{\sum }1\left(s_{i}\neq N\right)}$
F1_1_	$\frac{2\cdot \text{Precision}{}_{1}\cdot \text{Recall}}{\text{Precision}{}_{1}+\text{Recall}}$
F1_2_	$\frac{2\cdot \text{Precision}{}_{2}\cdot \text{Recall}}{\text{Precision}{}_{2}+\text{Recall}}$

## 6. Experimental Results

### 6.1. Collaborative Data Relabeling

#### 6.1.1. Comparative Experimental Results

**Figure 6:**
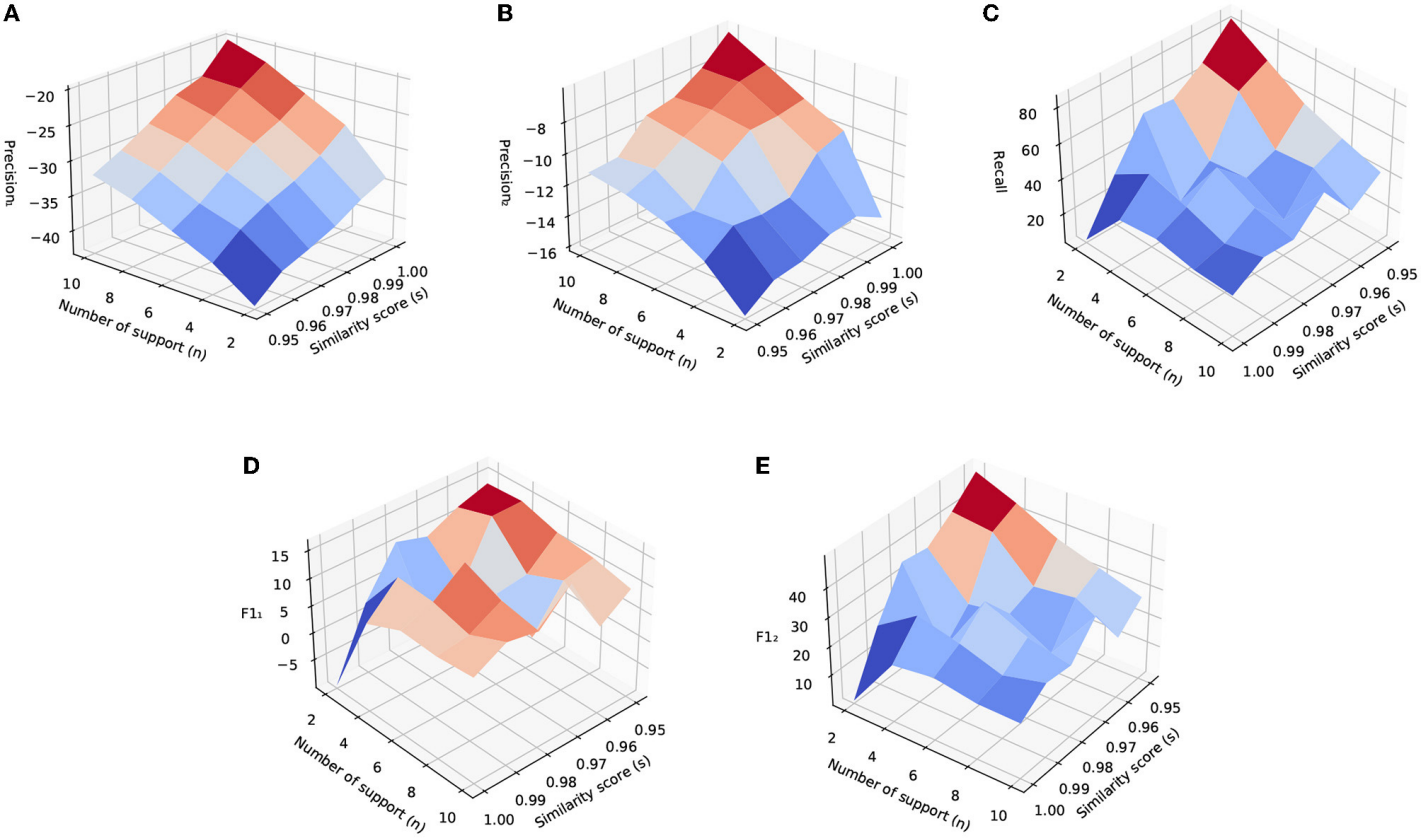
Experimental results of CDR method by varying the number of support and similarity threshold. These results are using similarity scores based on utterance embedding vectors generated by fine-tuned BERT encoder. The unit of the vertical axis is the percentage. **(A)** Precision^1^. **(B)** Precision^2^. **(C)** Recall. **(D)** F1_1_. **(E)** F1_2_.

Figure 6 shows the relative performance improvements of the CDR method with the change of hyperparameters, the number of support *n* and similarity threshold *s*. The baseline model is a listwise reranker model trained with the original training data. Figures 6A,B show precision_1_ and precision_2_ with respect to *n* and *s*. They show that precision_1_ and precision_2_ increase with the increasing of *n* and *s*. With the increasing of *s*, the relabels we obtain are from closer neighbors which tend to bring cleaner labels. When the similarity score is lower, the two utterances are less similar, which even leads to wrong labels. With the increasing of *n*, we require more neighbors to confirm the relabeling of a skill candidate, which leads to the higher quality of labels. Relatively, the precision of the model decreases with respect to the baseline models. The reason is that after relabeling, the model's recommendations are more diverse, which hurts the accuracy of the model as suggested by Castagnos et al. (2013) etc. For a more fair comparison of how diversity can impact user satisfaction, we manually compare the two models and the results are shown in Section 6.1.2.

Figure 6C shows the change of recall with respect to *n* and *s*. It shows that by lowering *s* and *n*, recall increases roughly. When lowering *s* and *n*, we bring more positive labels, therefore, the model is more likely to make a suggestion. Figures 6D,E show F1_1_ and F1_2_. From them, we can see that the overall performance of the model trained on relabeled data is higher than that of the baseline model.

#### 6.1.2. Manual Evaluation

To evaluate how CDR impacts the model performance, we manually compare the relabeled model against the model trained on original data. We randomly sampled 2500 samples and asked human annotators to check the suggested skills by relabeled and baseline models. We use two types of evaluation metrics. The first one is accuracy which is the number of correct predictions divided by the total number of predictions. As the model only makes a suggestion if the predicted probability is higher than 0.5, the model can reject to make a suggestion if it is not confident enough. To compare no suggestion with the suggestion, we use a score. A model gets a score by the following rules:

- If the model's prediction is correct, it gets a score.
- If the model does not make a suggestion: a) if the other model makes a wrong prediction, the current model gets a score; b) if the other model makes a correct prediction, the current model does not get a score. The intuition is that no suggestion is better than a wrong suggestion and a correct suggestion is better than no suggestion.

Table 2 shows the evaluation results based on manual annotation. The baseline model is the model trained on original data. From the results, we can see that the relabeled model has higher accuracy even though the offline metrics show the opposite, which indicates higher user satisfaction. The relabeled model also gets a higher score and makes more suggestions.

**Table 2:** Manual annotation results.

**Model**	**Accuracy**	**Score**	**#suggestions**
CDR-based model	+19.21%	+62.73%	+112.13%

#### 6.1.3. The Influence of Similarity Measurements

We experiment with several similarity measurements between utterances as discussed in Section 3.4, including Jaccard similarity, TF-IDF, and semantic similarity. Table 3 shows the comparative performance of models using different similarity measurements. By F1_1_ and F1_2_, we can see that relabeling by using semantic similarity achieves the best result. TF-IDF performs the worst as it only considers term matching which ignores the semantic meaning of an utterance. Jaccard similarity utilizes the skill candidates generated by the shortlister which is also based on term matching, therefore, it performs worse than semantic similarity. However, even the worst relabeling method outperforms the baseline model in terms of F1_1_ and F1_2_, which shows the efficacy of CDR.

**Table 3:** The influence of different similarity measurements on performance.

**Model**	**Precision_1_**	**Precision_2_**	**Recall**	**F1_1_**	**F1_2_**
Jaccard similarity	–32.54%	-12.35%	+43.31%	+9.21%	+27.53%
TF-IDF	–10.39%	–4.75%	+5.94%	+0.854%	+3.69%
Semantic similarity	–42.93%	–16.09%	+85.06%	+13.98%	+49.83%

### 6.2. Rephrase-Based Relabeling

Table 4 shows the relative performance change of rephrase-based relabeling with respect to the baseline. The baseline model is the listwise reranker trained on original data. The relabeled model is the listwise reranker model trained on the data with rephrase-based relabeling. A total of 4% of the training utterances are relabeled by using the label of the rephrased utterances. From the table, we can see that rephrase-based relabeling can improve both precision and recall. As rephrase-based relabeling brings more positive labels, the model trained on relabeled data is more likely to suggest skills, which drives the recall higher. In addition, rephrase-based relabeling corrects wrong labels, which improves precision.

**Table 4:** Experimental results on rephrase-based relabeling.

**Model**	**Precision_1_**	**Precision_2_**	**Recall**	**F1_1_**	**F1_2_**
Rephrase-based model	+2.22%	+4.45%	+10.67%	+5.54%	+8.09%

### 6.3. Ablation Study

In this section, we study how different features and components of the model impact the model performance. Specifically, we study the influence of skill id, skill name, skill score, and Bi-LSTM layer on model performance. We remove one of the factors while fixing the others. Table 5 shows the experimental results. From the results, we can see that removing skill id results in higher precisions and lower recall and F1 scores. Removing skill name and skill score increases precisions slightly and decreases recall and F1 scores. Removing Bi-LSTM layer drops recall significantly and leads to much lower F1 scores. In summary, skill id and Bi-LSTM layer have the most impact on model performance.

**Table 5:** Experimental results of ablation study.

**Model**	**Precision_1_**	**Precision_2_**	**Recall**	**F1_1_**	**F1_2_**
Without skill id	+9.71%	+1.45%	–28.5%	–20.83%	–24.13%
Without skill name	+0.82%	+0.05%	–2.83%	–1.84%	–2.27%
Without skill score	+0.15%	+0.15%	–0.55%	–0.35%	–0.41%
Without Bi-LSTM layer	+11.35%	+1.35%	–52.46%	–43.46%	–46.97%

### 6.4. Online Experiments

After seeing performance gains in offline experiments, we put FROST into online A/B testing. We compare FROST with the rule-based heuristic model. The online experiments show that FROST reduced the friction rate by 0.35%. Friction means the circumstances where the voice assistant does not understand the user and cannot act on the user's request. The number of accepted skills increased by 5.86%. The average number of new skills enabled per customer increased by 0.98%. Skill has to be enabled before it can be used by the customer. In addition, the number of unique suggested and accepted skills increased by 233 and 98.75%, respectively, which indicates that the new model makes more diverse suggestions than the legacy system.

## 7. Related Work

With more and more content created online, people are suffering from information overload. To help people find interesting information, recommender systems have been proposed and are a popular research topic in both academia and industry. Recommender systems have been widely adopted in the industry for recommending videos, music, books, etc. They are an integral part of many online services.

The earliest recommender systems are based on collaborative filtering or content-based filtering. Collaborative filtering assumes that similar users have similar interests and recommends items from similar users (Sarwar et al., 2001). Content-based filtering recommends items similar to the items the user likes (Aggarwal, 2016). Basilico and Hofmann (2004) unify collaborative filtering and content-based filtering by using a suitable kernel function between user-item pairs. The critical part of collaborative filtering and content-based filtering is similarity measurements. Similarities between users or items can be learned or calculated based on user or item features. Ning and Karypis (2011) proposed to learn the item similarity matrix by using sparse linear models. Another line of work for recommender systems is based on matrix factorization (Hu et al., 2008; Koren et al., 2009). Matrix factorization methods decompose the user-item rating matrix into user and item embedding vectors. The predicted rating of a user-item pair is the dot product of their corresponding user and item embedding vectors. Different variants of matrix factorization methods have been proposed such as non-negative matrix factorization (Zhang et al., 2006), SVD++ (Koren, 2008), timeSVD++ (Koren, 2009), and factorization machines (Rendle, 2010). Xiao et al. (2018) proposed a matrix factorization model for recommending music in IPAs. To cope with the limitation of VUI, they binarize play durations to obtain implicit affinity labels. Rendle et al. (2012) proposed Bayesian personalized ranking (BPR) which directly optimizes a ranking measure with the assumption that users prefer observed items over non-observed items. With this assumption, BPR naturally deals with missing and negative observations.

Deep learning has been more and more popular in many fields such as computer vision, natural language processing, etc. (LeCun et al., 2015). In the recommender system, we have also seen many works based on deep learning. Matrix factorization models user-item ratings using the inner product. Instead of using the inner product, neural collaborative filtering replaces it with a neural architecture (He et al., 2017). A deep learning version of the factorization machine, DeepFM, was proposed by Guo et al. (2017). DeepFM is an end-to-end model that can model both low- and high- order feature interactions. Covington et al. (2016) proposed two-stage deep learning models for Youtube video recommendation. To combine memorization and generalization, a wide and deep learning model was proposed for Google Play apps recommendation (Cheng et al., 2016). To model fashion evolution over time, one-class collaborative filtering (He and McAuley, 2016) has been proposed, which utilizes a time-window mechanism to highlight trends in a time window. Ma et al. (2020) proposed HRNN-meta to model temporal effects by encoding time information through a learned embedding.

The aforementioned studies focus on optimizing prediction accuracy. However, optimizing prediction accuracy is not necessarily optimizing user satisfaction. Ziegler et al. (2005) found that diversification of the recommendation topic can improve user satisfaction. Different methods have been proposed to improve the diversity of recommendations. Adomavicius and Kwon (2011) proposed a parameterized ranking approach to improve diversity. They also proposed a graph-theoretic approach to increase the diversity of recommended items based on maximum bipartite matching computations. Premchaiswadi et al. (2013) proposed a total diversity effect ranking for improving diversity by considering the diversity effect of each item in the recommendation list. Sun et al. (2020) proposed a recommendation method based on Bayesian graph convolutional neural networks. The node-copying model in their study can promote recommendation diversity.

The partial observation problem discussed in this article is similar to position bias in the ranking and recommender system. Position bias refers to the phenomenon that users are more likely to interact with items in a higher position of the recommendation list (Chen et al., 2020). Joachims et al. (2017) found that users are less likely to browse items that are ranked lower in the list, while only examining the top few items with eyetracking. Compared to position bias, the partial observation problem studied in this study is more severe as only the first item is exposed to the user.

## 8. Conclusions and Future Work

In this study, we proposed FROST, a skill recommender system to suggest skills for unhandled voice commands in IPAs that aims to reduce user friction and recover the conversation. Compared to traditional recommender systems, skill recommender systems face the challenges of partial observation, noisy labels, and hard and noisy utterances. To solve these challenges, we proposed two relabeling techniques, i.e., CDR and rephrase-based relabeling. CDR mitigates partial observation and noisy label problems. In addition, it improves the diversity of recommended skills. CDR as a simple and effective approach is especially useful for industrial deployment. We also developed rephrase-based relabeling method to overcome the hard and noisy utterance problem. We evaluated the proposed system offline before putting it online for A/B testing. The online experimental results showed significant gains in user experience metrics. In the future, we will try contextual bandits and let the model learn to explore the unsuggested skills.

## Data Availability Statement

The datasets presented in this article are not readily available because the data is private. Requests to access the datasets should be directed to QH, huqia@amazon.com.

## Author Contributions

QH is responsible for collaborative data relabeling and its corresponding experiments, and also writes most of the article. TM is responsible for working on rephrase based relabeling and related experiments. WX, XG, ZG, and RA reviewed the article. XM pulled the data and reviewed the article. MA reviewed the paper and preparing the data. All authors contributed to the article and approved the submitted version.

## Conflict of Interest

All authors were employed by Amazon.com.

## Publisher's Note

All claims expressed in this article are solely those of the authors and do not necessarily represent those of their affiliated organizations, or those of the publisher, the editors and the reviewers. Any product that may be evaluated in this article, or claim that may be made by its manufacturer, is not guaranteed or endorsed by the publisher.
